# Efficacy and safety for combination of t-EMG with O-arm assisted pedicle screw placement in neurofibromatosis type I scoliosis surgery

**DOI:** 10.1186/s13018-021-02882-9

**Published:** 2021-12-20

**Authors:** Xiexiang Shao, Zifang Huang, Jingfan Yang, Yaolong Deng, Junlin Yang, Wenyuan Sui

**Affiliations:** 1 Spine Center, Xin Hua Hospital Affiliated To Shanghai Jiao Tong University School of Medicine, No. 1665 Kongjiang Road, Shanghai, People’s Republic of China; 2 Department of Spine Surgery, Sun Yat-Sen University First Affiliated Hospital, No. 58 Second Zhongshan Road, Guangzhou, Guangdong People’s Republic of China

**Keywords:** NF-1, O-arm-assist, Pedicle screw, Triggered screw electromyography

## Abstract

**Background:**

Due to the characteristics of neurofibromatosis type I (NF-1) scoliosis, the precise placement of pedicle screws still remains to be a challenge. Triggered screw electromyography (t-EMG) has been proved to exhibit high sensitivity to identify mal-positioned pedicle screws, but no previous study assessed the combination of t-EMG with O-arm-assisted pedicle screw placement in NF-1 scoliosis surgery.

**Objective:**

To evaluate efficacy and safety for combination of t-EMG with O-arm-assisted pedicle screw placement in NF-1 scoliosis surgery.

**Materials and methods:**

From March 2018 to April 2020, sixty-five NF-1 scoliosis patients underwent t-EMG and O-arm-assisted pedicle screw fixation were retrospectively reviewed. The channel classification system was applied to classify the pedicle morphology based on pedicle width measurement by preoperative computed tomography scans. The minimal t-EMG threshold for screw path inspection was used as 8 mA, and operative screw redirection was also recorded. All pedicle screws were verified using a second intraoperative O-arm scan. The correlation between demographic and clinical data with amplitude of t-EMG were also analyzed.

**Results:**

A total of 652 pedicle screws (T10-S1) in 65 patients were analyzed. The incidence of an absent pedicle (channel classification type C or D morphology) was 150 (23%). Overall, abnormal t-EMG threshold was identified in 26 patients with 48 screws (7.4%), while 16 out of the 48 screws were classified as G0, 14 out of the 48 screws were classified as G1, and 18 out of the 48 screws were classified as G2. The screw redirection rate was 2.8% (18/652). It showed that t-EMG stimulation detected 3 unacceptable mal-positioned screws in 2 patients (G2) which were missed by O-arm scan. No screw-related neurological or vascular complications were observed.

**Conclusions:**

Combination of t-EMG with O-arm-assisted pedicle screw placement was demonstrated to be a safe and effective method in NF-1 scoliosis surgery. The t-EMG could contribute to detecting the rupture of the medial wall which might be missed by O-arm scan. Combination of t-EMG with O-arm could be recommended for routine use of screw insertion in NF-1 scoliosis surgery.

## Introduction

Scoliosis resulting from neurofibromatosis type I (NF-1) accounts for 2% of pediatric scoliosis [1]. Most severe NF-1 scoliosis is accompanied by greatly destroyed vertebrae and pedicle [2]. Thus, pedicle screw placement is quite challenging in NF-1 scoliosis surgery [3]. Mal-position rate of pedicle screws may reach 40% in the thoracolumbar spine, and there was approximately 1% of neurological complication rate [4]. Furthermore, potential biomechanical weakness of the instrumentation could contribute to implant failure or pseudarthrosis [5]. Thus, techniques which ensure the safety and increase the position accuracy of pedicle screws in NF-1 scoliosis should be emphasized.

Intra-operative O-arm-based spinal navigation can improve accuracy of initial pedicle screws position, and further help detect and reposition the mal-positioned screws [6, 7]. However, it has been reported that screw mal-position still inevitably occurred in NF-1 scoliosis even using intraoperative O-arm scanning [8]. Triggered screw electromyography (t-EMG) is a well-established intraoperative monitoring technique which helps identify mal-positioned screws. Thus, t-EMG is currently gaining increased popularity in spinal deformity surgery [9, 10]. However, several investigators were skeptical for solely use of t-EMG due to its false negatives/positives and differed thresholds [10–14].

Thus, the trend to maximize the safety of pedicle screw placement encouraged us to combine t-EMG with O-arm scan. We conduct the current retrospective study to verify efficacy and safety of t-EMG in combination with O-arm-assisted pedicle screw placement in NF-1 scoliosis surgery. The hypothesis of current study is that combination of t-EMG with O-arm-assisted pedicle screw placement was a safe and effective method in NF-1 scoliosis surgery.

## Materials and methods

### Patients

After obtaining approval from our institutional review board, consecutive eligible patients diagnosed with NF-1 scoliosis were retrospectively reviewed. All the patients underwent posterior spinal fusion involving thoracolumbar spine (T10–S1) using t-EMG and O-arm-assisted pedicle screw placement. No exclusion criteria were applied for gender, age, clinical or medical condition. A preoperative low dose 64-slice CT scan with slice thickness of 2 mm was applied to classify the pedicle morphology according to the channel classification system [15]. Type C or D morphology were defined as abnormality. In addition, Cobb angle was measured, and location of redirection screws were also recorded. Periapical area was defined as three vertebral levels above and below the apical vertebra.

### O-arm-assist technique

After midline exposure, the reference frame was fixed to the spinous process, and the binocular infrared camera were adjusted to receive the reflector ball. The first 3D scan was acquired in 13 s, and the intraoperative 3D images were then automatically obtained and visualized on O-arm viewing station (Stealth Station S7 Navigation System; Medtronic, Minneapolis, MN, USA). With the guidance of virtual navigation images, pedicle screws were routinely placed after verifying the integrity of medial wall. Once all screws have been placed, a second intraoperative 3D scan with O-arm were conducted to confirm whether they were in the target position.

### Triggered EMG

Abdominal muscles were used to verify the position of thoracic screws, and lower extremity muscles (iliopsoas, hip adductors, quadriceps and tibialis anterior) were selected to verify the position of lumbar screws. The target muscles tested in t-EMG were as follows: lower rectus abdominis for T10, T11; inferior rectus abdominis for T12, L1; vastus medialis and adductor magnus for L1-L4; vastus lateralis and tibialis anterior for L4, L5; proneous longus and gastrocnemius for L5, S1. A pulse width of 200 us and a repetition rate of 3.0 Hz were set for pedicle screw stimulation, and a monopolar electrode was used for t-EMG. A total intravenous anesthesia technique with no neuromuscular blockade was used throughout the procedures.

### Assessment of the pedicle screws

Pedicle screw violations were categorized into four grades according to Laine’s classification: grade 0 (G0), screws were completely within the pedicle; grade 1(G1), penetration less than 2 mm; grade 2 (G2), penetration between 2 and 4 mm; and grade 3 (G3), penetration over 4 mm [16]. Screws classified as G0 and G1 were considered as acceptable, while screws classified as G2 and G3 were considered unacceptable which should be repositioned. Intraoperative direction of breach was established by tactile palpation using a flexible ball-tipped metal probe.

### Statistical analysis

SPSS 17.0 for Windows (SPSS Inc., Chicago, IL, USA) was used for statistical verification, and *p* value < 0.05 was defined as statistically significant. Data were presented as mean ± standard deviation. Logistic regression analysis was performed between demographic or clinical parameters (age, gender, curve magnitude and pedicle width) and t-EMG amplitude.

## Results

**Fig. 1:**
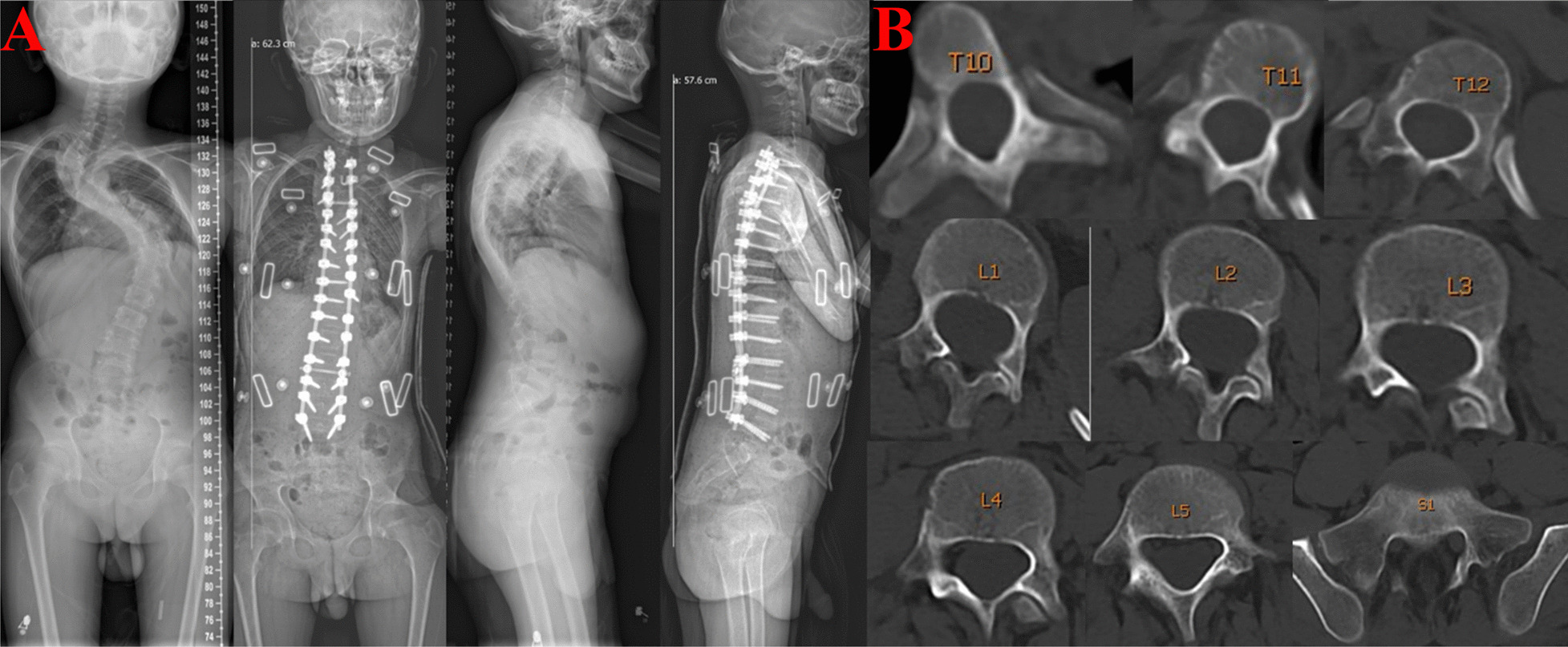
Representative preoperative and immediate postoperative images for neurofibromatosis type 1 (NF-1) scoliosis. **A** The 13-year-old patient obtained significantly improvement both in coronal and sagittal spinal deformity after undergoing posterior correction with t-EMG and O-arm-assisted pedicle screw fixation. **B** Routine preoperative CT indicated extremely thin pedicles in apical region

Sixty-five patients (24 males, 41 females) with mean age of 16.2 ± 7.3 years (range 13–24 years) were enrolled (Table 1). The major curve was corrected from 97.3 ± 4.6° to 26.8 ± 9.3° immediately after operation, representing an overall correction rate of 71.8 ± 3.7% (Fig. 1). A total of 652 pedicle screws (T10-S1) were analyzed. The incidence of an absent pedicle (channel classification type C or D morphology) was 23% (150/652), while 77% (502/652) pedicles were classified as channel Type A and B. Overall, abnormal t-EMG was identified in 26 patients with 48 screws (7.4%), while 16 out of the 48 screws were classified as G0, 14 out of the 48 screws were classified as G1, and 18 out of the 48 screws were classified as G2. All the 18 screws classified as G2 were located in the periapical area of the curve.

**Table1:** Clinical parameters of participants

Variable	Value
Age (years)	14.49 ± 1.74
Male /Female	24/41
Preoperative cobb angle (degrees)	97.3 ± 4.6
Postoperative cobb angle (degrees)	26.8 ± 9.3
Correction rate (%)	71.8 ± 3.7
Total pedicle screws (T10-S1)	652
Pedicle width (mm)	3.68 ± 0.57
Pedicle morphology	
A	370
B	132
C	108
D	42
Abnormal t-EMG	48
Sensitivity (%) Specificity (%) Positive predictive value (%)	100 96.2 66.7
Screw position of abnormal t-EMG G0 G1 G2 G3	16 14 18 0
Screws requiring redirection	18
Redirection rate (%)	2.8
Dural tear	1
Screw-related neuro-complication	0

In addition, there were 3 unacceptable mal-positioned screws (G2) in 2 patients detected by t-EMG stimulation but missed by O-arm scan. All of the 3 screws were located in the periapical area of the curve. When used in combination with O-arm, t-EMG was found to obtain a sensitivity of 100%, specificity of 96.2%, and a positive predictive value of 66.7%. Logistic regression analysis showed there was positive correlation between t-EMG amplitude and pedicle width (Table 2).

**Table 2:** Relationships between parameters and t-EMG amplitude analyzed by logistic regression

Variable	*P* value	95% confidence interval
Age	0.414	0.284–1.388
Gender	0.432	0.078–3.802
Curve magnitude	0.236	0.998–1.266
Pedicle width	0.004	0.030–0.623

For complications, dural tear occurred in 1 patient during operation due to bony spur from broken pedicle medial wall. No additional screw-related neurological or vascular complications were observed.

## Discussion

Although pedicle screws provide numerous advantages, placement of pedicle screws in NF-1 scoliosis still remains a challenge since the vertebrae and pedicle are always seriously destroyed, especially in the apical region [15, 17]. Misplaced pedicle screw fixation would contribute to neurological deficits and potential biomechanical weakness of the instrumentation, which further results in implant failure or pseudarthrosis [18–20]. Thus, the importance of safe and accurate insertion of pedicle screws should be highlighted, and techniques which ensure safety and increase the accurate rate of pedicle screws placement are always the focus of research.

Silbermann et al. noted that higher accurate rate of pedicle screw could be obtained in O-arm group than that in free-hand group [21]. It was also reported that intra-operative O-arm-based navigation technique could decrease the incidence of pedicle screw misplacement in scoliosis surgery when compared to traditional fluoroscopy [6, 22, 23]. However, Jin et al. showed that although 90.2% of pedicle screws could be accurately placed with O-arm navigation system, NF-1 scoliosis patients using O-arm assisted technique intraoperatively were still at high risk of pedicle screw misplacement [8]. In our study, O-arm-assisted screw placement technique were applied to insert a total of 652 pedicle screws (T10-S1). Twenty-nine screws (7.4%) were identified abnormal by a second O-arm scan in 26 patients, in which 15 screws were classified as G2, 14 screws were classified as G1. Three screws (G2) in 2 patients were missed by a second O-arm scan. Two reasons might account for it. First, osseous structural destruction of the apical region, rotation of vertebral column and partial overlap of anatomical structures led O-arm difficult to accurately identify the anatomical junction, and further failed to identify the relationship between screws and medial wall of the pedicle. Second, a difference between virtual and intra-operative pedicle screws, which has been described in lumber spine surgeries, might be generated in NF-1 scoliosis assisted by O-arm navigation [24].

The t-EMG was developed as a method to electrically stimulate the positioned pedicle screw to assess its proximity to nearby nerve roots. However, t-EMG was criticized for its false negatives and positives, and thresholds utilized for safety assessment differed in the thoracic and lumbar regions according to previous published literature [10, 14]. Samdani et al. suggested that t-EMG is not reliable in detecting medial breaches from T2 to T9 [10]. Alemos et al. set 8 mA as stimulation threshold, and 3 false negative EMGs in 3 patients were detected postoperatively by new neurologic deficits [11]. Mavrogenis et al. concluded that 7 mA stimulation threshold had a 98.73% positive predictive value for accurate pedicle screw placement [12]. In addition, Mikula et al. reviewed 18 studies with 15,065 screws in 2932 patients and found overall sensitivity of t-EMG was 0.78, and the specificity was 0.94 utilizing threshold criteria from the individual studies [13]. The authors found that the 10–12 mA threshold had the greatest reported receiver operating characteristic area under the curve, with a sensitivity of 0.82 and specificity of 0.97. In current study, our electrophysiologist alerts us with threshold of 8 mA, and then these screws are removed and checked. Forty-eight screws (7.4%) have been identified with abnormal t-EMG threshold in 26 patients, of which 18 screws were classified as G2, 14 screws were classified as G1, 16 screws were classified as G0. t-EMG was found to have a sensitivity of 100%, specificity of 96.2%, with a positive predictive value of 66.7%. This may be due to the thin pedicle in the parietal vertebral region, which is dominated by type C and D, while the medial wall became thinner after screw placement, which leads to a higher sensitivity. In our experience, 8 mA was used as the alarm threshold, and the obtained t-EMG value was compared with the adjacent vertebral pedicles, which might improve the sensitivity of t-EMG. Of course, this procedure also partly depends on the experience of the electrophysiologist.

In current study, the combination of O-arm and t-EMG was applied to assist pedicle screw placement in NF-1 scoliosis. All the broken medial wall could be effectively recognized during operation, and no neurological complications caused by screw misplacement occurred. Although limitations exist in individual technique, we should acknowledge that combination of two techniques greatly contributes to the accurate pedicle screw placement and avoiding the occurrence of neurological complications in IF-1 scoliosis. However, due to the limitation of sample size, the results of this study still need to be verified with a larger sample.

## Conclusions

Combination of t-EMG with O-arm-assisted pedicle screw placement was demonstrated to be a safe and effective method in NF-1 scoliosis surgery. The t-EMG could contribute to detecting the rupture of the medial wall which might be missed by O-arm scan. Combination of t-EMG with O-arm could be recommended for routine use of screw insertion in NF-1 scoliosis surgery.

## Data Availability

All the data and materials are available by sending an e-mail to the corresponding author.

## Abbreviations

NF-1: Neurofibromatosis type I
t-EMG: Triggered screw electromyography
